# (1α,8β)-6β-Benzo­yloxy-6-dehydroxy­heteratisine from *Aconitum zeravschanicum*

**DOI:** 10.1107/S1600536809023873

**Published:** 2009-06-27

**Authors:** Bakhodir Tashkhodjaev, Bakhodir T. Salimov

**Affiliations:** a S.Yunusov Institute of Chemistry of Plant Substances, Academy of Sciences of Uzbekistan, M. Ulugbek Str. 77, Tashkent 100170, Uzbekistan

## Abstract

The title compound, C_29_H_37_NO_6_, was isolated from *Aconitum zeravschanicum* and exhibits anti­arhythmic activity. It is a derivative of the diterpenoid alkaloid heteratisine and as such the core framework of the mol­ecule contains four six-membered, three seven-membered and one five-membered ring. The chair conformation of one of the meth­oxy-substituted six-membered rings is different from that observed in heteratisine hydro­bromide monohydrate. In the latter case, this ring adopts a boat conformation due to a stabilizing intra­molecular N—H⋯O hydrogen bond. In the crystal structure of the title compound, there is only one acidic H atom. This hydroxyl group forms an inter­molecular O—H⋯O hydrogen bond that links mol­ecules into infinite chains along the *b* axis.

## Related literature

For the isolation and idenfication of 6-benzoyl­heteratisine, see: Aneja *et al.* (1973 ▶), Jacobs *et al.* (1947 ▶), Nigmatullaev *et al.* (2000 ▶). For anti­arhythmic activity, see: Salimov *et al.* (1996 ▶). For the structure of heteratisine hydro­bromide monohydrate, see: Przybylska (1965 ▶).
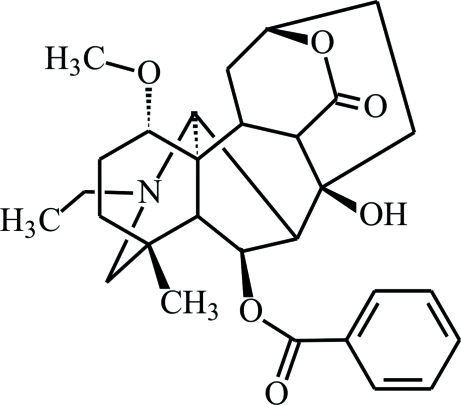


## Experimental

### 

#### Crystal data

C_29_H_37_NO_6_
*M* *_r_* = 495.60
Orthorhombic, 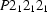

*a* = 10.039 (5) Å
*b* = 14.107 (8) Å
*c* = 17.512 (6) Å
*V* = 2480 (2) Å^3^
*Z* = 4
Mo *K*α radiation
μ = 0.09 mm^−1^
*T* = 300 K
0.50 × 0.30 × 0.15 mm

#### Data collection

Stoe Stadi-4 four-circle diffractometer
Absorption correction: none
2481 measured reflections
2481 independent reflections
1667 reflections with *I* > 2σ(*I*)
3 standard reflections every 200 reflections intensity decay: 6.8%

#### Refinement

*R*[*F* ^2^ > 2σ(*F* ^2^)] = 0.067
*wR*(*F* ^2^) = 0.142
*S* = 1.22
2481 reflections
330 parameters
H-atom parameters constrained
Δρ_max_ = 0.22 e Å^−3^
Δρ_min_ = −0.21 e Å^−3^

### 

Data collection: *STADI4* (Stoe & Cie, 1997 ▶); cell refinement: *STADI4*; data reduction: *X-RED* (Stoe & Cie, 1997 ▶); program(s) used to solve structure: *SHELXS97* (Sheldrick, 2008 ▶); program(s) used to refine structure: *SHELXL97* (Sheldrick, 2008 ▶); molecular graphics: *SHELXTL* (Sheldrick, 2008 ▶); software used to prepare material for publication: *SHELXTL*.

## Supplementary Material

Crystal structure: contains datablocks I, global. DOI: 10.1107/S1600536809023873/zl2222sup1.cif

Structure factors: contains datablocks I. DOI: 10.1107/S1600536809023873/zl2222Isup2.hkl

Additional supplementary materials:  crystallographic information; 3D view; checkCIF report

## Figures and Tables

**Table 1:** Hydrogen-bond geometry (Å, °)

*D*—H⋯*A*	*D*—H	H⋯*A*	*D*⋯*A*	*D*—H⋯*A*
O3—H3⋯O1^i^	0.82	2.25	3.056 (8)	166

Symmetry code: (i) 
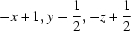
.

**Fig. 1.**
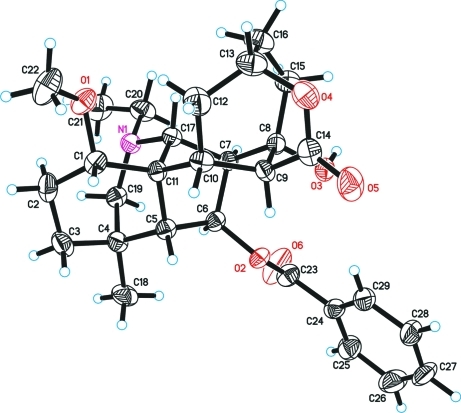
The molecular structure of 6-benzoylheteratisine, showing the atomic numbering scheme and displacement ellipsoids drawn at the 30% probability level.

**Fig. 2.**
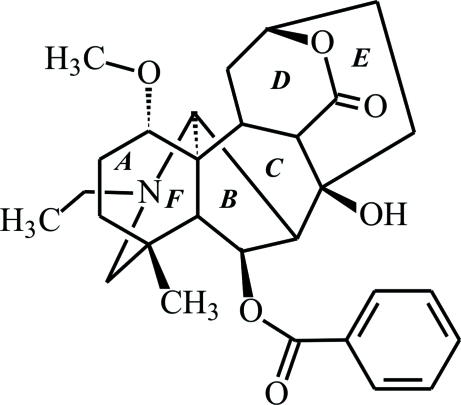
Ring assigments in 6-benzoylheteratisine.

**Fig. 3.**
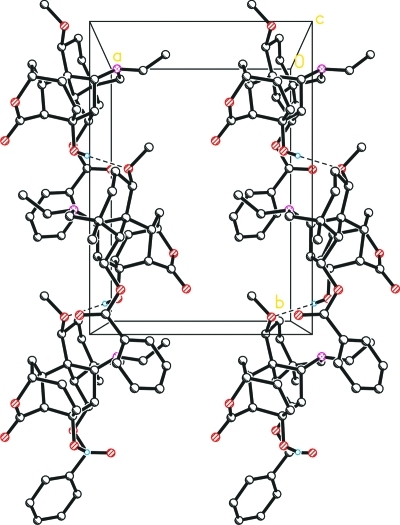
View along the c direction of the crystal packing of 6-benzoylheteratisine, showing the formation of hydrogen bonds (dashed lines). H-atoms not involved in hydrogen bonding have been removed for clarity.

## supplementary crystallographic information

### Comment

The title compound of this study, 6-benzoylheteratisine, C_29_H_37_NO_6_, was first obtained synthetically in 1973 (Aneja *et al.*, 1973) and found to be a derivative of a naturally occuring compound (Jacobs *et al.* 1947). Later it was isolated from *Aconitum zeravschanicum* Steinb (Nigmatullaev *et al.* 2000). 6-Benzoylheteratisine exibits antiarhythmic activity that exceeds other antiarrhythmic drugs of the quinidine groups (Salimov *et al.* 1996). The crystal structure of the parent compound was previously established as a salt in the form of heteratisine hydrobromide monohydrate (Przbylslka, 1965).

The molecular structure of the title compound is shown in Fig. 1. The heteratisine skeleton contains four six-membered rings, (*A*, *C*, *D* and *F*), one five-membered ring (*B*), and three seven-membered rings (e.g. *E*, others not labeled for clarity) (Fig. 2). Ring *B* has an envelope and ring *C*a more or less regular chair conformation. Ring *F* shows a significant distortion and rings *D* and *E* adopt a boat conformations. The chair conformation of ring *A* in the title molecule is different from that observed in heteratisine hydrobromide monohydrate (Przybylska, 1965). For the salt of the parent compound ring *A* adopts a boat conformation due to a stabilizing intramolecular N—H···O hydrogen bond between the protonated amine towards the oxygen atom, an interaction not present in the title compound.

The aromatic ring and the acyl-group are rotated against each other, the dihedral angle of their respective planes is 32.6 (9)°. There is only one acidic hydrogen atom in the crystal structure of the title compound. This hydroxyl group forms an intermolecular O—H···O hydrogen bond that links the molecules into infinite chains along the *b-*axis. (Table 1; Fig.3)

### Experimental

The title compound was isolated from the chloroform fraction of the leaves of *Aconitum zeravschanicum* by a known method (Nigmatullaev *et al.*, 2000). Crystals suitable for X-ray analysis were obtained by slow evaporation of an ethanol solution at room temperature (m.p. 485–487 K).

### Refinement

The hydroxyl H atom was located in a difference Fourier map but was ultimately placed geometrically (with an O—H distance of 0.82 Å). The H atoms bonded to C atoms were placed geometrically (with C—H distances of 0.98 Å for CH; 0.97 Å for CH_2_; 0.96 Å for CH_3_; and 0.93 Å for C_ar_) and included in the refinement in a riding motion approximation with U_iso_=1.2U_eq_(C) [U_iso_=1.5U_eq_(C,O) for methyl and hydroxyl H atoms].

### Crystal data

**Table:**

C_29_H_37_NO_6_	*D*_x_ = 1.327 Mg m^−^^3^
*M**_r_* = 495.60	Melting point: 486(2) K
Orthorhombic, *P*2_1_2_1_2_1_	Mo *K*α radiation, λ = 0.71073 Å
Hall symbol: P 2ac 2ab	Cell parameters from 30 reflections
*a* = 10.039 (5) Å	θ = 10–20°
*b* = 14.107 (8) Å	µ = 0.09 mm^−^^1^
*c* = 17.512 (6) Å	*T* = 300 K
*V* = 2480 (2) Å^3^	Prizmatic, colourless
*Z* = 4	0.50 × 0.30 × 0.15 mm
*F*(000) = 1064	

### Data collection

**Table:**

Stoe Stadi-4 four-circle diffractometer	*R*_int_ = 0.0000
Radiation source: fine-focus sealed tube	θ_max_ = 25.0°, θ_min_ = 1.9°
graphite	*h* = 0→11
Scan width (ω) = 1.56 – 1.68, scan ratio 2θ:ω = 1.00 I(Net) and sigma(I) calculated according to Blessing (1987)	*k* = 0→16
2481 measured reflections	*l* = 0→20
2481 independent reflections	3 standard reflections every 200 reflections
1667 reflections with *I* > 2σ(*I*)	intensity decay: 6.8%

### Refinement

**Table:**

Refinement on *F*^2^	Secondary atom site location: difference Fourier map
Least-squares matrix: full	Hydrogen site location: inferred from neighbouring sites
*R*[*F*^2^ > 2σ(*F*^2^)] = 0.067	H-atom parameters constrained
*wR*(*F*^2^) = 0.142	*w* = 1/[σ^2^(*F*_o_^2^) + (0.0342*P*)^2^ + 1.8083*P*] where *P* = (*F*_o_^2^ + 2*F*_c_^2^)/3
*S* = 1.22	(Δ/σ)_max_ = 0.001
2481 reflections	Δρ_max_ = 0.22 e Å^−^^3^
330 parameters	Δρ_min_ = −0.20 e Å^−^^3^
0 restraints	Extinction correction: *SHELXL97* (Sheldrick, 2008), Fc^*^=kFc[1+0.001xFc^2^λ^3^/sin(2θ)]^-1/4^
Primary atom site location: structure-invariant direct methods	Extinction coefficient: 0.0056 (10)

### Special details

**Table:**

Geometry. All e.s.d.'s (except the e.s.d. in the dihedral angle between two l.s. planes) are estimated using the full covariance matrix. The cell e.s.d.'s are taken into account individually in the estimation of e.s.d.'s in distances, angles and torsion angles; correlations between e.s.d.'s in cell parameters are only used when they are defined by crystal symmetry. An approximate (isotropic) treatment of cell e.s.d.'s is used for estimating e.s.d.'s involving l.s. planes.
Refinement. Refinement of *F*^2^ against ALL reflections. The weighted *R*-factor *wR* and goodness of fit *S* are based on *F*^2^, conventional *R*-factors *R* are based on *F*, with *F* set to zero for negative *F*^2^. The threshold expression of *F*^2^ > σ(*F*^2^) is used only for calculating *R*-factors(gt) *etc*. and is not relevant to the choice of reflections for refinement. *R*-factors based on *F*^2^ are statistically about twice as large as those based on *F*, and *R*- factors based on ALL data will be even larger.

### Fractional atomic coordinates and isotropic or equivalent isotropic displacement parameters (Å^2^)

**Table:**

	*x*	*y*	*z*	*U*_iso_*/*U*_eq_	
O1	0.6593 (4)	0.5430 (3)	0.3119 (3)	0.0562 (12)	
O2	0.6307 (4)	0.1410 (3)	0.4078 (2)	0.0416 (10)	
O3	0.6046 (4)	0.1087 (3)	0.2572 (2)	0.0520 (12)	
H3	0.5411	0.0905	0.2317	0.062*	
O4	0.8674 (5)	0.2617 (4)	0.1608 (3)	0.0634 (13)	
O5	0.9303 (5)	0.1410 (4)	0.2317 (3)	0.0797 (17)	
O6	0.4463 (5)	0.0591 (4)	0.4323 (4)	0.099 (2)	
N1	0.4096 (5)	0.4035 (4)	0.3632 (3)	0.0437 (13)	
C1	0.6830 (6)	0.4820 (4)	0.3766 (4)	0.0473 (17)	
H1A	0.7785	0.4850	0.3879	0.057*	
C2	0.6104 (7)	0.5245 (5)	0.4445 (4)	0.062 (2)	
H2A	0.6567	0.5813	0.4609	0.075*	
H2B	0.5212	0.5426	0.4290	0.075*	
C3	0.6020 (7)	0.4566 (5)	0.5099 (4)	0.062 (2)	
H3A	0.5491	0.4844	0.5505	0.074*	
H3B	0.6906	0.4446	0.5297	0.074*	
C4	0.5388 (6)	0.3628 (5)	0.4847 (3)	0.0456 (16)	
C5	0.6284 (6)	0.3133 (4)	0.4264 (3)	0.0413 (15)	
H5A	0.7135	0.2947	0.4495	0.050*	
C6	0.5531 (6)	0.2269 (4)	0.3963 (3)	0.0416 (15)	
H6A	0.4708	0.2207	0.4259	0.050*	
C7	0.5146 (6)	0.2506 (4)	0.3142 (3)	0.0385 (14)	
H7A	0.4242	0.2279	0.3037	0.046*	
C8	0.6122 (6)	0.2102 (4)	0.2542 (3)	0.0420 (15)	
C9	0.7520 (6)	0.2353 (4)	0.2812 (3)	0.0417 (15)	
H9A	0.7690	0.1973	0.3271	0.050*	
C10	0.7671 (6)	0.3403 (4)	0.3048 (3)	0.0436 (16)	
H10A	0.8465	0.3426	0.3373	0.052*	
C11	0.6509 (6)	0.3782 (4)	0.3553 (3)	0.0373 (14)	
C12	0.8015 (8)	0.3998 (5)	0.2341 (4)	0.068 (2)	
H12A	0.7441	0.4551	0.2330	0.082*	
H12B	0.8926	0.4220	0.2387	0.082*	
C13	0.7873 (7)	0.3479 (6)	0.1600 (4)	0.067 (2)	
H13A	0.8244	0.3890	0.1202	0.080*	
C14	0.8569 (7)	0.2086 (5)	0.2239 (4)	0.0540 (18)	
C15	0.5827 (7)	0.2387 (5)	0.1723 (4)	0.0566 (19)	
H15A	0.4868	0.2451	0.1678	0.068*	
H15B	0.6085	0.1859	0.1400	0.068*	
C16	0.6447 (7)	0.3279 (5)	0.1382 (4)	0.070 (2)	
H16A	0.6399	0.3232	0.0830	0.084*	
H16B	0.5909	0.3819	0.1533	0.084*	
C17	0.5155 (5)	0.3605 (4)	0.3181 (3)	0.0377 (14)	
H17A	0.5149	0.3869	0.2664	0.045*	
C18	0.5181 (7)	0.3020 (5)	0.5555 (3)	0.066 (2)	
H18C	0.6006	0.2968	0.5829	0.098*	
H18D	0.4886	0.2400	0.5406	0.098*	
H18E	0.4522	0.3309	0.5877	0.098*	
C19	0.4026 (6)	0.3801 (5)	0.4445 (3)	0.0461 (16)	
H19A	0.3485	0.3235	0.4504	0.055*	
H19B	0.3572	0.4313	0.4708	0.055*	
C20	0.2808 (6)	0.3924 (5)	0.3252 (4)	0.061 (2)	
H20A	0.2541	0.3264	0.3277	0.073*	
H20B	0.2904	0.4091	0.2718	0.073*	
C21	0.1728 (7)	0.4528 (6)	0.3602 (5)	0.081 (3)	
H21A	0.0972	0.4544	0.3267	0.122*	
H21B	0.2057	0.5161	0.3676	0.122*	
H21C	0.1470	0.4265	0.4085	0.122*	
C22	0.7553 (7)	0.6160 (5)	0.3047 (5)	0.082 (3)	
H22A	0.7518	0.6418	0.2540	0.123*	
H22B	0.8425	0.5905	0.3140	0.123*	
H22C	0.7368	0.6651	0.3411	0.123*	
C23	0.5638 (7)	0.0621 (5)	0.4222 (4)	0.0532 (18)	
C24	0.6515 (7)	−0.0215 (4)	0.4266 (4)	0.0470 (16)	
C25	0.6195 (8)	−0.0936 (5)	0.4745 (4)	0.065 (2)	
H25A	0.5420	−0.0897	0.5034	0.078*	
C26	0.7004 (10)	−0.1734 (6)	0.4811 (5)	0.083 (3)	
H26A	0.6796	−0.2213	0.5156	0.100*	
C27	0.8113 (10)	−0.1796 (5)	0.4357 (5)	0.082 (3)	
H27A	0.8664	−0.2325	0.4388	0.099*	
C28	0.8405 (8)	−0.1089 (5)	0.3865 (5)	0.073 (2)	
H28A	0.9148	−0.1147	0.3551	0.087*	
C29	0.7625 (7)	−0.0279 (5)	0.3814 (4)	0.0565 (18)	
H29A	0.7851	0.0209	0.3481	0.068*	

### Atomic displacement parameters (Å^2^)

**Table:**

	*U*^11^	*U*^22^	*U*^33^	*U*^12^	*U*^13^	*U*^23^
O1	0.046 (3)	0.045 (2)	0.077 (3)	−0.004 (2)	−0.001 (3)	0.009 (2)
O2	0.040 (2)	0.036 (2)	0.050 (3)	0.002 (2)	0.005 (2)	0.008 (2)
O3	0.057 (3)	0.043 (2)	0.056 (3)	0.003 (2)	−0.005 (2)	−0.008 (2)
O4	0.062 (3)	0.075 (3)	0.053 (3)	−0.001 (3)	0.014 (3)	−0.005 (3)
O5	0.058 (3)	0.093 (4)	0.088 (4)	0.023 (3)	0.005 (3)	−0.024 (4)
O6	0.051 (3)	0.059 (3)	0.187 (6)	−0.004 (3)	0.018 (4)	0.014 (4)
N1	0.039 (3)	0.049 (3)	0.043 (3)	0.009 (3)	0.000 (3)	−0.003 (3)
C1	0.038 (3)	0.043 (4)	0.061 (4)	−0.003 (3)	−0.005 (3)	0.002 (4)
C2	0.058 (4)	0.049 (4)	0.080 (5)	0.009 (4)	−0.005 (4)	−0.023 (4)
C3	0.063 (5)	0.072 (5)	0.050 (4)	0.011 (4)	−0.003 (4)	−0.022 (4)
C4	0.039 (3)	0.057 (4)	0.041 (4)	0.013 (3)	0.002 (3)	−0.007 (3)
C5	0.036 (3)	0.044 (3)	0.044 (4)	0.004 (3)	0.003 (3)	0.001 (3)
C6	0.036 (3)	0.043 (4)	0.046 (4)	0.006 (3)	0.004 (3)	0.000 (3)
C7	0.038 (3)	0.038 (3)	0.040 (3)	−0.004 (3)	−0.003 (3)	0.000 (3)
C8	0.045 (4)	0.037 (3)	0.044 (4)	0.002 (3)	0.000 (3)	−0.005 (3)
C9	0.042 (3)	0.047 (4)	0.036 (3)	0.003 (3)	0.001 (3)	−0.008 (3)
C10	0.033 (3)	0.054 (4)	0.044 (4)	−0.003 (3)	−0.002 (3)	0.003 (3)
C11	0.033 (3)	0.039 (3)	0.040 (3)	0.003 (3)	0.003 (3)	−0.003 (3)
C12	0.078 (5)	0.068 (5)	0.058 (5)	−0.013 (4)	0.023 (4)	0.001 (4)
C13	0.066 (5)	0.082 (6)	0.053 (5)	−0.005 (5)	0.011 (4)	0.012 (4)
C14	0.038 (4)	0.062 (5)	0.062 (5)	0.002 (4)	−0.005 (4)	−0.013 (4)
C15	0.056 (4)	0.062 (4)	0.052 (4)	0.002 (4)	−0.007 (4)	0.005 (4)
C16	0.066 (5)	0.093 (6)	0.050 (4)	0.000 (5)	0.005 (4)	0.006 (4)
C17	0.033 (3)	0.045 (3)	0.036 (3)	0.003 (3)	0.001 (3)	0.000 (3)
C18	0.070 (5)	0.089 (6)	0.038 (4)	0.010 (5)	0.011 (4)	0.002 (4)
C19	0.040 (3)	0.049 (4)	0.049 (4)	0.006 (3)	0.007 (3)	0.002 (3)
C20	0.041 (4)	0.087 (6)	0.053 (4)	0.012 (4)	−0.005 (4)	0.000 (4)
C21	0.053 (5)	0.094 (6)	0.097 (7)	0.024 (5)	0.001 (5)	0.004 (6)
C22	0.061 (5)	0.050 (4)	0.136 (8)	−0.014 (4)	0.012 (6)	0.010 (5)
C23	0.049 (4)	0.046 (4)	0.064 (5)	−0.006 (4)	−0.002 (4)	0.006 (4)
C24	0.056 (4)	0.036 (3)	0.050 (4)	−0.006 (3)	−0.009 (4)	0.005 (3)
C25	0.076 (5)	0.051 (4)	0.068 (5)	−0.004 (5)	0.004 (5)	0.004 (4)
C26	0.106 (7)	0.063 (6)	0.081 (6)	0.002 (5)	−0.017 (6)	0.021 (5)
C27	0.108 (7)	0.041 (4)	0.099 (7)	0.023 (5)	−0.042 (6)	0.003 (5)
C28	0.073 (5)	0.066 (5)	0.079 (5)	0.028 (5)	−0.021 (5)	−0.017 (5)
C29	0.068 (5)	0.043 (4)	0.059 (4)	−0.003 (4)	−0.013 (4)	−0.004 (4)

### Geometric parameters (Å, °)

**Table:**

O1—C22	1.416 (7)	C10—H10A	0.9800
O1—C1	1.443 (7)	C11—C17	1.528 (8)
O2—C23	1.325 (7)	C12—C13	1.496 (9)
O2—C6	1.454 (7)	C12—H12A	0.9700
O3—C8	1.434 (7)	C12—H12B	0.9700
O3—H3	0.8200	C13—C16	1.509 (10)
O4—C14	1.339 (8)	C13—H13A	0.9800
O4—C13	1.458 (9)	C15—C16	1.527 (9)
O5—C14	1.213 (8)	C15—H15A	0.9700
O6—C23	1.194 (8)	C15—H15B	0.9700
N1—C17	1.457 (7)	C16—H16A	0.9700
N1—C20	1.463 (7)	C16—H16B	0.9700
N1—C19	1.463 (7)	C17—H17A	0.9800
C1—C2	1.518 (8)	C18—H18C	0.9600
C1—C11	1.544 (8)	C18—H18D	0.9600
C1—H1A	0.9800	C18—H18E	0.9600
C2—C3	1.495 (9)	C19—H19A	0.9700
C2—H2A	0.9700	C19—H19B	0.9700
C2—H2B	0.9700	C20—C21	1.509 (9)
C3—C4	1.532 (9)	C20—H20A	0.9700
C3—H3A	0.9700	C20—H20B	0.9700
C3—H3B	0.9700	C21—H21A	0.9600
C4—C18	1.523 (8)	C21—H21B	0.9600
C4—C5	1.529 (8)	C21—H21C	0.9600
C4—C19	1.556 (8)	C22—H22A	0.9600
C5—C6	1.529 (8)	C22—H22B	0.9600
C5—C11	1.562 (8)	C22—H22C	0.9600
C5—H5A	0.9800	C23—C24	1.473 (9)
C6—C7	1.526 (8)	C24—C25	1.357 (9)
C6—H6A	0.9800	C24—C29	1.370 (9)
C7—C8	1.546 (8)	C25—C26	1.392 (10)
C7—C17	1.552 (8)	C25—H25A	0.9300
C7—H7A	0.9800	C26—C27	1.372 (12)
C8—C15	1.518 (8)	C26—H26A	0.9300
C8—C9	1.523 (8)	C27—C28	1.351 (10)
C9—C14	1.504 (8)	C27—H27A	0.9300
C9—C10	1.545 (8)	C28—C29	1.388 (9)
C9—H9A	0.9800	C28—H28A	0.9300
C10—C12	1.536 (8)	C29—H29A	0.9300
C10—C11	1.559 (8)		
			
C22—O1—C1	113.0 (5)	O4—C13—C12	110.3 (6)
C23—O2—C6	117.1 (4)	O4—C13—C16	111.7 (6)
C8—O3—H3	109.5	C12—C13—C16	113.7 (7)
C14—O4—C13	115.6 (5)	O4—C13—H13A	106.9
C17—N1—C20	110.7 (5)	C12—C13—H13A	106.9
C17—N1—C19	117.9 (5)	C16—C13—H13A	106.9
C20—N1—C19	112.1 (5)	O5—C14—O4	119.0 (7)
O1—C1—C2	107.5 (5)	O5—C14—C9	123.2 (7)
O1—C1—C11	110.0 (5)	O4—C14—C9	117.7 (6)
C2—C1—C11	117.6 (5)	C8—C15—C16	120.6 (6)
O1—C1—H1A	107.1	C8—C15—H15A	107.2
C2—C1—H1A	107.1	C16—C15—H15A	107.2
C11—C1—H1A	107.1	C8—C15—H15B	107.2
C3—C2—C1	111.9 (5)	C16—C15—H15B	107.2
C3—C2—H2A	109.2	H15A—C15—H15B	106.8
C1—C2—H2A	109.2	C13—C16—C15	116.3 (6)
C3—C2—H2B	109.2	C13—C16—H16A	108.2
C1—C2—H2B	109.2	C15—C16—H16A	108.2
H2A—C2—H2B	107.9	C13—C16—H16B	108.2
C2—C3—C4	110.9 (5)	C15—C16—H16B	108.2
C2—C3—H3A	109.5	H16A—C16—H16B	107.4
C4—C3—H3A	109.5	N1—C17—C11	110.5 (4)
C2—C3—H3B	109.5	N1—C17—C7	115.8 (5)
C4—C3—H3B	109.5	C11—C17—C7	100.8 (5)
H3A—C3—H3B	108.1	N1—C17—H17A	109.8
C18—C4—C5	111.5 (5)	C11—C17—H17A	109.8
C18—C4—C3	107.9 (5)	C7—C17—H17A	109.8
C5—C4—C3	110.0 (5)	C4—C18—H18C	109.5
C18—C4—C19	109.7 (5)	C4—C18—H18D	109.5
C5—C4—C19	106.7 (5)	H18C—C18—H18D	109.5
C3—C4—C19	111.0 (5)	C4—C18—H18E	109.5
C4—C5—C6	107.7 (5)	H18C—C18—H18E	109.5
C4—C5—C11	110.4 (5)	H18D—C18—H18E	109.5
C6—C5—C11	105.3 (5)	N1—C19—C4	115.6 (5)
C4—C5—H5A	111.1	N1—C19—H19A	108.4
C6—C5—H5A	111.1	C4—C19—H19A	108.4
C11—C5—H5A	111.1	N1—C19—H19B	108.4
O2—C6—C7	116.6 (5)	C4—C19—H19B	108.4
O2—C6—C5	110.6 (5)	H19A—C19—H19B	107.4
C7—C6—C5	106.0 (5)	N1—C20—C21	112.9 (6)
O2—C6—H6A	107.8	N1—C20—H20A	109.0
C7—C6—H6A	107.8	C21—C20—H20A	109.0
C5—C6—H6A	107.8	N1—C20—H20B	109.0
C6—C7—C8	113.6 (5)	C21—C20—H20B	109.0
C6—C7—C17	100.1 (5)	H20A—C20—H20B	107.8
C8—C7—C17	113.3 (5)	C20—C21—H21A	109.5
C6—C7—H7A	109.8	C20—C21—H21B	109.5
C8—C7—H7A	109.8	H21A—C21—H21B	109.5
C17—C7—H7A	109.8	C20—C21—H21C	109.5
O3—C8—C15	106.8 (5)	H21A—C21—H21C	109.5
O3—C8—C9	105.6 (5)	H21B—C21—H21C	109.5
C15—C8—C9	114.3 (5)	O1—C22—H22A	109.5
O3—C8—C7	108.0 (5)	O1—C22—H22B	109.5
C15—C8—C7	114.9 (5)	H22A—C22—H22B	109.5
C9—C8—C7	106.6 (5)	O1—C22—H22C	109.5
C14—C9—C8	112.3 (5)	H22A—C22—H22C	109.5
C14—C9—C10	110.5 (5)	H22B—C22—H22C	109.5
C8—C9—C10	113.4 (5)	O6—C23—O2	123.9 (7)
C14—C9—H9A	106.7	O6—C23—C24	123.7 (7)
C8—C9—H9A	106.7	O2—C23—C24	112.3 (6)
C10—C9—H9A	106.7	C25—C24—C29	120.0 (7)
C12—C10—C9	109.3 (5)	C25—C24—C23	119.3 (7)
C12—C10—C11	116.0 (5)	C29—C24—C23	120.6 (6)
C9—C10—C11	114.0 (5)	C24—C25—C26	121.3 (8)
C12—C10—H10A	105.5	C24—C25—H25A	119.4
C9—C10—H10A	105.5	C26—C25—H25A	119.4
C11—C10—H10A	105.5	C27—C26—C25	118.5 (8)
C17—C11—C1	116.3 (5)	C27—C26—H26A	120.8
C17—C11—C10	111.6 (5)	C25—C26—H26A	120.8
C1—C11—C10	107.8 (5)	C28—C27—C26	119.9 (8)
C17—C11—C5	96.6 (5)	C28—C27—H27A	120.0
C1—C11—C5	113.2 (5)	C26—C27—H27A	120.0
C10—C11—C5	111.1 (5)	C27—C28—C29	121.8 (8)
C13—C12—C10	114.3 (6)	C27—C28—H28A	119.1
C13—C12—H12A	108.7	C29—C28—H28A	119.1
C10—C12—H12A	108.7	C24—C29—C28	118.4 (7)
C13—C12—H12B	108.7	C24—C29—H29A	120.8
C10—C12—H12B	108.7	C28—C29—H29A	120.8
H12A—C12—H12B	107.6		

### Hydrogen-bond geometry (Å, °)

**Table:**

*D*—H···*A*	*D*—H	H···*A*	*D*···*A*	*D*—H···*A*
O3—H3···O1^i^	0.82	2.25	3.056 (8)	166

Symmetry codes: (i) −*x*+1, *y*−1/2, −*z*+1/2.
